# Leukocyte and cytokine variables in asymptomatic Pugs at genetic risk of necrotizing meningoencephalitis

**DOI:** 10.1111/jvim.16293

**Published:** 2021-10-23

**Authors:** Rebecca Windsor, Samuel D. Stewart, Joshua Talboom, Candace Lewis, Marcus Naymik, Ignazio S. Piras, Stefan Keller, Dori L. Borjesson, Gary Clark, Chand Khanna, Matthew Huentelman

**Affiliations:** ^1^ Ethos Veterinary Health Woburn Massachusetts USA; ^2^ Ethos Discovery (501c3) San Diego California USA; ^3^ Neurogenomics Division Translational Genomics Research Institute Phoenix Arizona USA; ^4^ Department of Pathology, Microbiology, Immunology University of California, Davis Davis California USA; ^5^ Gary Clark Statistical Consulting LLC Superior Colorado USA

**Keywords:** cytokine, genetic risk, immune dysregulation, necrotizing meningoencephalitis

## Abstract

**Background:**

Necrotizing meningoencephalitis (NME, aka Pug dog encephalitis) is an inflammatory brain condition associated with advanced disease at initial presentation, rapid progression, and poor response to conventional immunomodulatory therapy.

**Hypothesis/Objectives:**

That genetic risk for NME, defined by a common germline DNA haplotype located on chromosome 12, is associated with altered blood cytokine concentrations and leukocyte subsets in asymptomatic Pugs.

**Animals:**

Forty Pug dogs asymptomatic for NME from a hospital sample.

**Methods:**

Prospective observational cohort study, including germline genome‐wide genotyping, plasma cytokine determination by multiplexed profiling, and leukocyte subset characterization by flow cytometric analysis.

**Results:**

Seven (18%) dogs were high risk, 10 (25%) medium risk, and 23 (58%) low risk for NME, giving a risk haplotype frequency of 30%. High and medium risk Pugs had significantly lower proportion of CD4+ T cells (median 22% [range, 7.3%‐38%] vs 29% [range, 16%‐41%], *P* = .03) and higher plasma IL‐10 concentrations than low‐risk Pugs (median 14.11 pg/mL [range, 9.66‐344.19 pg/mL] vs 12.21 pg/mL [range, 2.59‐18.53 pg/mL], *P* = .001). No other variables were significantly associated with the NME haplotype‐based risk.

**Conclusions and Clinical Importance:**

These data suggest an immunological underpinning to NME and a biologic rationale for future clinical trials that investigate novel diagnostic, preventative, and therapeutic strategies for this disease.

**Abbreviations**
CNS: central nervous system
CSF: cerebrospinal fluid
DLA II: dog leukocyte antigen
IFNγ: interferon gamma
IL: interleukin
MSC: mesenchymal stem cells
NME: necrotizing meningoencephalitis
PDE: Pug dog encephalitis

## 1 INTRODUCTION

Necrotizing meningoencephalitis (NME), first characterized in the 1980s,^1^ is a nonsuppurative, noninfectious encephalopathy that typically affects smaller breed dogs including the Maltese, Chihuahua, Yorkshire Terrier, Pekingese, and French Bulldog.^2^, ^3^, ^4^ The most common breed afflicted by NME is the Pug. It is estimated that NME accounts for 69%‐81% of all intracranial disease in this breed, leading to the development of the term Pug dog encephalitis (PDE).^5^ Disease onset in Pugs is most commonly between 1.5 and 2.5 years of age.^5^, ^6^

Clinical signs of NME include, but are not restricted to, lethargy, behavioral changes, seizures, circling, and visual deficits. Diagnosis is achieved through the combination of recognition of clinical signs, brain imaging via magnetic resonance imaging, cerebrospinal fluid (CSF) analysis, and ruling out infectious diseases that appear clinically similar. Histopathological examination, which is generally only available postmortem, reveals white and gray matter necrosis and thickening of the meninges because of infiltration and aggregation of lymphocytes, plasma cells, and macrophages.

There are genetic risk factors for NME^2^, ^6^, ^7^, ^8^ The strongest genetic risk factor in Pug dogs is located on chromosome 12 within the dog leukocyte antigen (DLA II) locus, although 2 additional genetic associations of importance within the IL7R and FBXW7 genes, also known as regulators of immune system function, are present in toy breeds.^2^ Of note, MHC II and IL7R are genes that harbor risk‐associated variants for neuroinflammatory diseases in humans, including multiple sclerosis.^9^, ^10^, ^11^ The well‐characterized genetic susceptibility, narrow age window of disease onset, and well‐defined clinical presentation suggest an opportunity to study pathogenesis of the disease and to assess the potential for immunomodulatory strategies to prevent NME.

## 2 MATERIALS AND METHODS

### 2.1 Dog selection and blood collection

Pug‐phenotype dogs reported by their owners as not having clinical signs of NME (inclusive of behavioral changes, seizures, circling, and visual deficits) were invited to participate in a prospective observational cohort study (“Pug Clinic”). After informed owner consent was obtained, dogs were enrolled in the Pug Clinic study, which had received Animal Care and Use Committee approval (Animal Clinical Investigation ACUC, Chevy Chase, MD). Enrolled dogs underwent a complete physical and neurological examination by a Board‐certified veterinary Neurologist. Only Pugs without signs consistent with NME were eligible (no dogs were identified with signs of NME) and had 10 mL of whole blood collected. After collection, the blood was transferred to 2 tubes containing EDTA (for CBC, flow cytometric leukocyte phenotyping, plasma cytokines, and germline risk assessment), and 1 serum separator tube (for serum biochemistry). A urine sample was also collected via cystocentesis for routine urinalysis. Genotyping after completion of data collection confirmed Pug breed in all dogs in this prospective cohort.

### 2.2 Germline breed and risk score assessment

Genomic DNA was isolated from frozen anticoagulated whole blood collected in K_2_EDTA coated tubes (BD, Franklin Lakes, NJ) via magnetic bead‐based purification using the Chemagic 360 (PerkinElmer, Waltham, MA) and the DNA Blood 400 Kit H96 (PerkinElmer, catalog #: CMG‐1091). Briefly, whole blood was thawed on ice, gently mixed, and 400 μL was pipetted into individual wells of a 96‐deep‐well plate. Protease (PerkinElmer, part of the kit: catalog #: CMG‐1091) was then added to each well before being processed on the Chemagic 360 using the 96‐rod head according to the manufacturer's protocol. DNA was eluted in ~150 μL of Elution Buffer (PerkinElmer) and concentration and quality were assessed. The average DNA yield reported via NanoDrop One (ThermoFisher, Waltham, MA) was 65.9 ng/μL, the average 260 : 280 wavelength ratio was 1.91, and the average 260 : 230 wavelength ratio was 2.40. Seven randomly selected DNA samples were further evaluated using Genomic Tape (Agilent, Santa Clara, CA) on the TapeStation 4200 instrument (Agilent). Each sample was considered high quality as the average DIN across the 7 samples was 8.93.

According to the manufacturer's protocol, approximately 200 ng of DNA from all samples was processed for genotyping using the CanineHD Whole‐Genome Genotyping BeadChip (Illumina, San Diego, CA). BeadChips were scanned on the iScanSystem (Illumina) to generate IDAT files. Genotype calling was carried out using GenomeStudio (Illumina) per the manufacturer's recommendations. The resulting genotype data was exported to PLINK format according to the CanFam3.1 assembly and was quality controlled to include SNPs with cross‐cohort missingness ≤95% and samples with a genotyping rate of ≥90%.

Principal components analysis was performed to identify nonpurebred dogs, and cohort‐wide relatedness was assessed using a pi‐hat metric to identify any cryptic relatedness (defined as a pi‐hat ≥0.2). After all quality control metrics were applied, no animals were removed from the analysis yielding a final cohort size of 40 animals. To identify an NME risk‐associated haplotype, we extracted SNPs from the Pug NME GWAS.^7^ We selected SNPs that were located within a 500 kb region centered on the top significant SNP association, BICF2P194998. This yielded a total of 25 SNPs each from 22 cases and 86 controls from Barber et al after selecting for samples and SNPs with a genotyping rate >95%, and SNPs with a Hardy‐Weinberg Equilibrium *P* > 1.0E−05, and MAF >0.05. Linkage disequilibrium (LD) blocks were estimated in this entire cohort of samples using Gabriel's method^12^ in Haploview v4.2.^13^ We identified an LD block of 146 kb encompassing 7 SNPs including the top Pug NME GWAS SNP, BICF2P194998 (Figure A in Supporting Information). After conducting haplotype association analysis in Haploview, we found 5 haplotypes within this LD block in the Barber et al cohort: 1 associated with increased NME risk (88.6% frequency in cases, 27.1% frequency in controls; *P* = 1.3E−13), and the remaining haplotypes were associated with decreased NME risk (Table A in Supporting Information). To assess NME risk in the study cohort detailed in this manuscript, we extracted the same 7 SNPs from the Illumina CanineHD BeadChip data and haplotypes were phased using the default options in Beagle 5.2.^14^ High‐risk animals were determined to be those that carried 2 risk haplotypes, medium‐risk animals carried 1 risk haplotype, and low‐risk animals carried no risk haplotypes.

### 2.3 Multiplexed plasma cytokine analysis

Anticoagulated whole blood (EDTA) was centrifuged at 3500*g* for 10 minutes at 4°C, after which the plasma was collected and stored at −80°C until analyzed. Cytokine concentrations were determined from plasma using a canine‐specific multiplex assay, following the manufacturer's instructions of diluting the plasma 3‐fold (CCYTOMAG‐90K, Millipore Sigma, Burlington, MA). The cytokine panel (7 analytes) was selected based on their potential role in inflammatory or immune pathways. The plasma dilutions were factored in for the reporting of the lower limits of detection and calculated analyte concentrations. Plasma concentrations of interferon gamma (IFNγ), interleukin (IL)‐2, IL‐6, IL‐10, IL‐15, monocyte chemoattractant protein‐1 (MCP‐1), and tumor necrosis factor α (TNFα) were determined (Luminex 200 System with xPONENT software, Luminex Corp, Austin, TX). Analyte concentrations were determined using a 5‐parameter logistic nonlinear regression curve, calculated from 7 standards, ranging from 2.44 to 10 000 pg/mL (IFNγ), or from 12.21 to 50 000 pg/mL (all other analytes). The actual lower limit of detection was based on the standard curve generated from the assay when the assays were run.

### 2.4 Leukocyte phenotyping

Leukocyte phenotypes of interest included CD4+ and CD8+ T lymphocytes, CD21+ B lymphocytes, and leukocyte activation phenotype including CD11b and MHCII expressing lymphocytes. Anticoagulated whole blood (EDTA; 40 μL) was diluted with 60 μL flow buffer (DPBS with 1% equine serum, 5 mM EDTA, and 0.01% NaN_3_). Equine serum was used as the flow buffer was formulated by the Leukocyte Antigen Biology Lab, UC Davis, for use with their antibodies and this formulation allowed for clean labeling of all antibodies when tested. Antibodies including 3 from the Leukocyte Antigen Biology Laboratory (CD3‐AF488 [CA17.2A12, 0.25 μL],^15^ CD4‐FITC [CA13.1E4, 1 μL],^16^ and CD8α‐PE [CA9.JD3, 1 μL]^16^), CD11b‐PE (M1/70, 0.1 μL, Invitrogen, Rockford, IL),^17^ CD25‐PE (P4A10, 10 μL, Bio‐Rad Laboratories, Hercules, CA), and MHC II‐PE (CVS20, 10 μL, Invitrogen) were added and allowed to incubate for 30 minutes at room temperature. After incubation, red blood cells were lysed using RBC Lysis Buffer (3 mL, 154 mM ammonium chloride, 10 mM potassium bicarbonate, 1 mM EDTA, pH 7.2) for 10 minutes at room temperature. Leukocytes were washed twice with flow buffer. Samples were interrogated with a FC500 flow cytometer using CXP software (Beckman Coulter, Miami, FL). Flow cytometric data were analyzed using FlowJo software (Becton Dickinson, San Jose, CA). All multicolor flow panels used fluorescence minus 1 (FMO) controls to compensate for color overlap. Gates were set such that background staining was less than 3%.

### 2.5 Statistical analyses of associations with NME haplotype‐based risk status

The percentages of dogs with detectable cytokines were compared between NME haplotype‐based risk (low risk vs high/moderate risk) using Fisher's exact tests. Then, undetectable values were replaced with the technical lower limit of detection for each cytokine and their distributions were tested for normality using Shapiro‐Wilk tests, Shapiro‐Francia tests, and tests for symmetry and kurtosis. The assumption of normality of the distribution was rejected for each of the cytokines by 1 or more of these tests; however, the assumption of normality was not rejected for the majority of the leukocyte phenotype distributions. For consistency, all subsequent analyses were performed using nonparametric statistics; comparisons between NME haplotype risk status using Wilcoxon rank sum tests.

All calculations were performed using Stata 14.2 (StataCorp. 2015, Stata Statistical Software: Release 14, College Station, TX: StataCorp LP). *P*‐values less than .05 were considered statistically significant. Because of the exploratory nature of this pilot study, no adjustments were made for multiple hypothesis testing.

## 3 RESULTS

The signalment (age, sex, and weight) of dogs recruited to the Pug Clinic are described in Table 1. No associations between signalment or weight and NME haplotype risk were found. No abnormalities were found in the CBC, serum chemistry, or urinalysis results of this eligible cohort (data not shown). No dogs were ineligible for inclusion.

**TABLE 1:** Signalment of Pug Clinic cohort

	All dogs (n = 40)
Sex	
Female (intact)	2 (5%)
Female (spayed)	14 (35%)
Male (intact)	2 (5%)
Male (neutered)	22 (55%)
Age	
Median (yr)	8
Range (yr)	1‐17
Weight^a^	
Median (kg)	9.5
Range (kg)	5.17‐14.2

^a^ Weights were unavailable for 9 dogs.

### 3.1 Haplotype‐based NME risk profile within the recruited cohort

**FIGURE 1:**
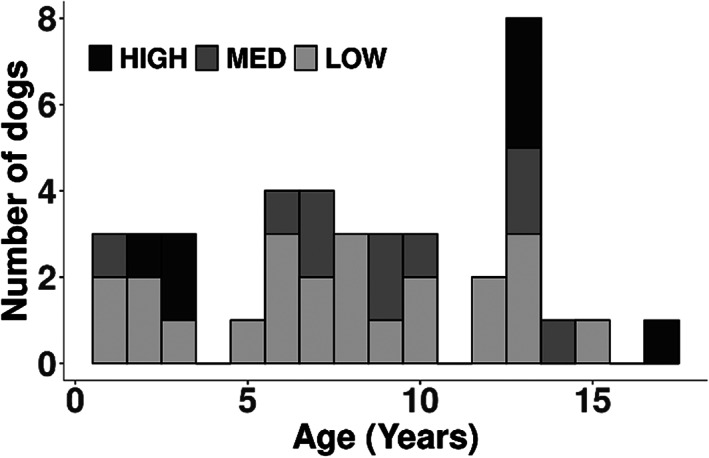
Age distribution among Pugs at low, medium, and high germline risk for necrotizing meningoencephalitis

Using the haplotype information obtained for each dog at the chromosome 12 NME risk locus^7^ low, medium, and high risk animals were identified across the entire age spectrum examined (1‐17 years of age, Figure 1). The rationale for defining haplotype risk was based on the number of risk haplotypes present in their germline, blood‐cell derived DNA wherein 0, 1, and 2 haplotypes were assigned a risk status of low, medium, or high, respectively. Within our recruited cohort of 40 dogs, 7 (18%) were high risk, 10 (25%) were medium risk, and 23 (58%) were low risk, resulting in a risk haplotype frequency of 30% in our prospective cohort.

### 3.2 Systemic inflammatory cytokine profiles assessment in the study cohort

Cytokines and leukocyte phenotypes in the peripheral blood of eligible dogs across the germline NME risk strata that were asymptomatic for NME were characterized. Cytokines were variably detected in plasma. Three dogs (8%) had no detectable cytokines, 16 dogs (40%) had only 1 or 2 cytokines detected, and the remaining 21 dogs (53%) had 3 or more cytokines detected in their plasma. IL‐15 was detected most frequently (33/40 dogs, 83%; range, 4.73‐86 848.02 pg/mL), followed by MCP‐1, detected in 24/40 (60%) of dogs (range, 180.46‐2398.74 pg/mL). All other cytokines were detected in less than 50% of the Pug dogs (Table 2).

**TABLE 2:** Detectable cytokine concentrations in study dogs stratified by germline risk for NME

Cytokine	NME genomic risk	N	Detectable	*P*‐value^a^	Median	Range	*P*‐value^b^
IFNγ	Low	23	5 (33%)	.72	6.13	1.6‐34.5	.6
	High/medium	17	5 (29%)		9.73	2.17‐163.25	
	Total	40	10 (25%)		6.58	1.6‐163.25	
IL‐2	Low	23	5 (23%)	.48	494.09	79.02‐1253.13	.58
	High/medium	17	6 (35%)		735.11	13.46‐31 587.96	
	Total	40	11 (23%)		494.09	13.46‐31 587.96	
IL‐6	Low	23	8 (33%)	.2	25.55	0.81‐391.01	.09
	High/medium	17	10 (59%)		307.24	5.33‐13 858.37	
	Total	40	18 (45%)		63.6	0.81‐13 858.37	
IL‐15	Low	23	20 (87%)	.43	44.69	4.73‐3588.98	.06
	High/medium	17	13 (73%)		201.54	9.72‐86 848.02	
	Total	40	33 (83%)		73.53	4.73‐86 848.02	
IL‐10	Low	23	8 (33%)	.2	5.11	2.59‐18.53	.001
	High/Medium	17	10 (59%)		58.99	9.66‐344.18	
	Total	40	18 (45%)		16.32	2.59‐344.18	
MCP‐1	Low	23	15 (65%)	.52	368.47	180.46‐670.74	.02
	High/medium	17	9 (53%)		539.78	363.23‐2398.74	
	Total	40	24 (60%)		400.84	180.46‐2398.74	
TNFα	Low	23	2 (9%)	.11	188.46	126.3‐250.63	.44
	High/medium	17	5 (29%)		430.69	34.28‐6657.1	
	Total	40	7 (18%)		250.63	34.28‐6657.1	

Abbreviations: IFNγ, interferon gamma; IL, interleukin; MCP‐1, monocyte chemoattractant protein‐1; NME, necrotizing meningoencephalitis; TNFα, tumor necrosis factor α.

^a^ *P*‐value from Fisher's exact test.

^b^ *P*‐value from Wilcoxon rank sum test.

### 3.3 Systemic inflammatory cytokine profiles assessment stratified by NME genomic risk

**FIGURE 2:**
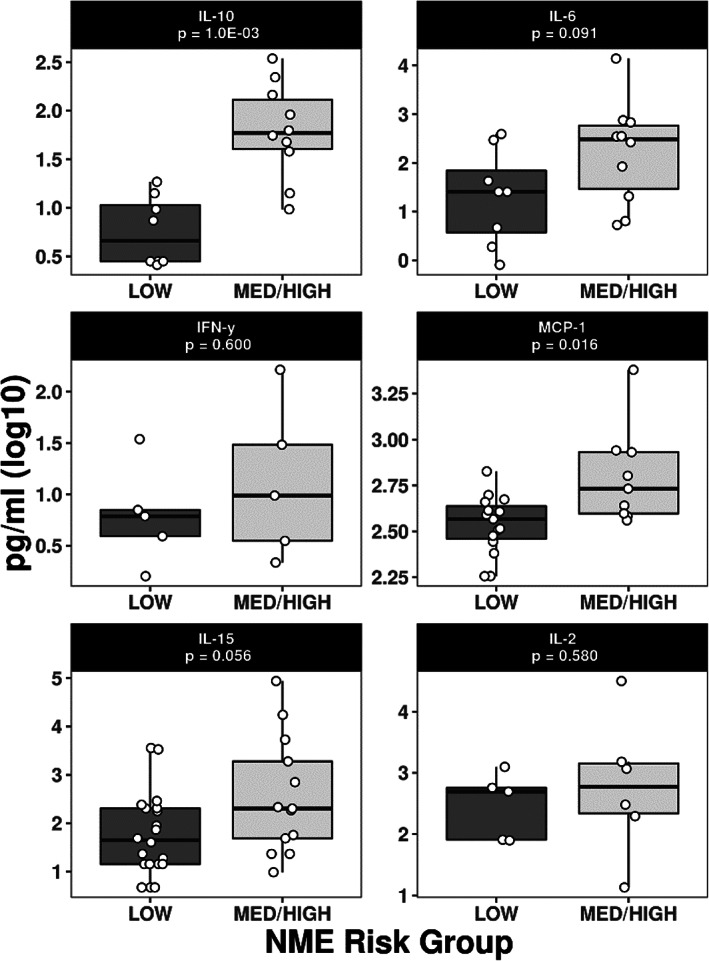
Cytokine concentration boxplots for dogs at low and medium/high germline risk for NME. Boxes represent the interquartile range from the 25th to 75th percentile with the median value indicated by the black horizontal line within the boxed portion. The bars show the range of the group data, with the white dots representing each dog's individual values. *P*‐values were calculated using nonparametric Wilcoxon rank tests on untransformed values. NME, necrotizing meningoencephalitis

There were 9 dogs that had a strong inflammatory cytokine profile (defined as ≥6/7 cytokines with abnormal high concentrations in plasma). Of these dogs, 6 were > 9 years of age and would largely be considered at low clinical risk for the development of NME. All 6 of these dogs had a low or medium risk haplotype and physical exam findings that are compatible with inflammatory profiles including obesity, cough (on immunosuppressive therapy), chronic ear infections, and caudal T3 myelopathy with paraparesis or moderate ataxia. Of the remaining 3 dogs, 2 were young (ages 2 and 3 years) and 1 was middle age (6 years). Of the young dogs, 1 was high risk, 1 was low risk. The high‐risk Pug had higher (and markedly increased) concentrations of IL‐2, IL‐6, IL‐10, IL‐15, MCP‐1, and TNFα compared to the 2 low risk and middle‐aged dogs. Among dogs with detectable cytokines, concentrations of IL‐10, MCP‐1, and IL‐15 were significantly greater in high/medium‐risk dogs compared with low‐risk dogs (*P* = .001, .02, and .06, respectively). However, when undetectable levels were replaced with the technical lower limit of detection, a distinctive systemic inflammatory response in dogs at‐risk for NME was only observed for IL‐10 (*P* = .001; Figure 2). This higher IL‐10 was not associated with other cytokine changes.

### 3.4 Leukocyte phenotype shifts assessment stratified by NME genomic risk

**FIGURE 3:**
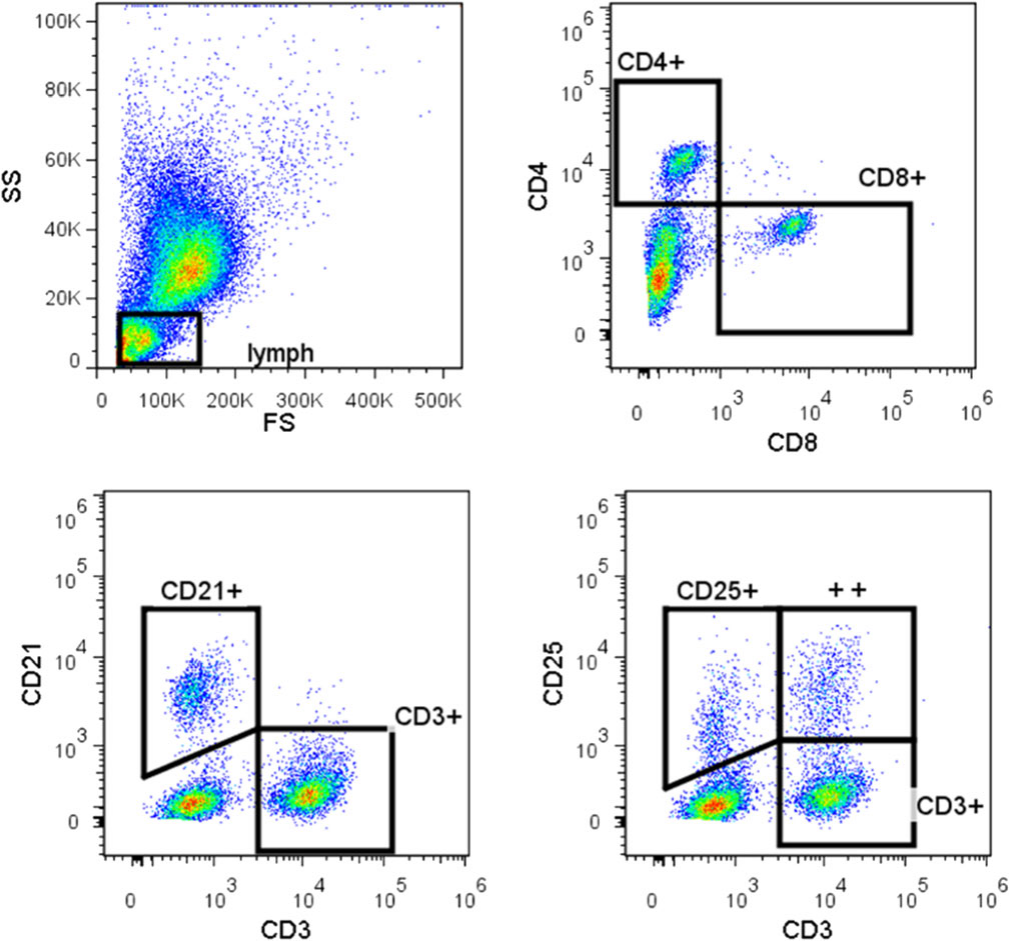
Flow gating strategy used for leukocyte phenotype shift assessment

**FIGURE 4:**
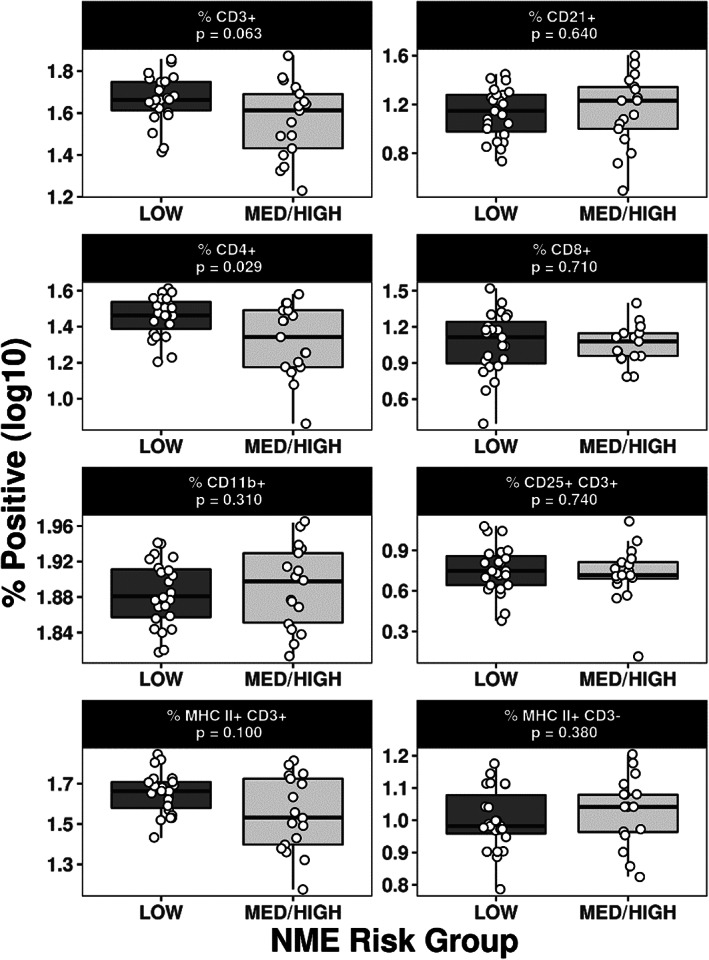
Leukocyte subset boxplots for dogs at low and medium/high germline risk for NME. Boxes represent the interquartile range from the 25th to 75th percentile with the median value indicated by the black horizontal line within the boxed portion. The bars show the range of the group data, with the white dots representing each dog's individual values. *P*‐values were calculated using nonparametric Wilcoxon rank sum tests. NME, necrotizing meningoencephalitis

The percentage of CD4+ T cells was significantly reduced in the dogs at high/medium risk compared to low risk for NME (*P* = .03; Figures 3 and 4). This reduction in CD4+ T cells was isolated and not associated with other significant changes in leukocyte subsets. There were no significant differences in CD8+ T cells (*P* = .71) or B cells (CD21+; *P* = .64) in dogs with high/medium risk compared to low‐risk Pug dogs.

The relatively small number of dogs in this study and the presence of collinearity and interdependence of some of the immune variables measured in this study, complicated our efforts to create a multivariate model of the immune variables that were independently associated with genomic risk for NME.

## 4 DISCUSSION

Our results suggest the presence of alterations in systemic immune variables in asymptomatic dogs at genetic risk for NME. Further studies are now needed to determine whether the observed changes in IL‐10 and CD4 are causally associated with the etiopathogenesis of NME and if there are immunomodulatory strategies that could be used to prevent or effectively treat PDE. Other alterations in immune variables that did not achieve statistical significance should not be ignored as part of future confirmatory studies (Table 2, Figure 2).

The cytokine IL‐10 is an important immune regulator that attenuates proinflammatory immune responses.^18^ Similarly CD4+ T cells are responsible for a cell‐mediated immune response. The relevance of this relatively immunosuppressed phenotype in the etiopathogenesis of NME remains unclear given the study limitations referenced above. Indeed, since this study did not include follow‐up, it is unknown if these altered immune variables are associated with the development of NME. For instance, it is conceivable, although unlikely, that only high‐risk dogs without these alterations would develop clinical disease. Nonetheless, these data provide a biological rationale to consider immunomodulation rather than immunosuppression as a management and prevention strategy for NME.

This study should be considered in the face of some limitations, 1 of which is the cohort composition and characteristics, which included both old and young dogs. The assessment of our exploratory findings would be improved by independent recruitment and replication of a larger‐sized asymptomatic cohort. Specifically, attention should be paid to the ages of the recruited animals, wherein a cohort more focused on animals below the median age of onset for NME would improve the opportunity to understand immune variable changes that could happen before the onset of the disease, whereas a cohort more populated by older animals could provide insight about how that immune system adaptations might be acquired to evade the genetic risk for NME. In our cohort, we recruited across a variety of ages to afford an examination under both scenarios because of the lack of information in general about the systemic immune state in the Pug dog and particularly in animals at varying genetic risk for NME. The recruited cohort was free of clinical signs consistent with NME, not all of the animals were rigorously screened to be free of all systemic and neurological conditions. It is possible that the presence of unidentified systemic conditions could alter the underlying immune system characteristics of the animal and could therefore positively or negatively confound the association with the immune system components that we profiled. Nonetheless, we did not find any evidence to suggest that systemic disease was present in this cohort of dogs. Furthermore, we are unaware of other consistent systemic inflammatory conditions to be associated with NME. An additional limitation is the variability in cytokine detection between dogs, which could impact the analysis of the cytokine data.

While the precise pathogenesis of different types of neuroinflammatory disorders remains to be elucidated, cytokines are vital in orchestrating the cellular components involved in initiating and sustaining neuroinflammation.^19^ In humans, classical neuroinflammatory, nondemyelinating diseases are triggered by the invasion of CNS tissues by blood‐borne leukocytes.^19^ In dogs, there are no studies on cytokine networks in neuroinflammation. To date, only a single study has assessed cytokine concentrations in Pugs with active NME and found markedly increased levels of IFNγ, but no consistent changes in the concentrations of IL‐4, IL‐10, IL‐17, or IL‐21.^20^

As noted above, our results of asymptomatic Pugs suggest immune dysregulation resulting from the altered at risk alleles or a secondary systemic consequence of subclinical neuroinflammation that “leaks” into the blood. The order direction and mutual exclusivity of these events cannot be resolved at this time. Marked infiltration of Th17‐producing CD163‐positive macrophages/microglia, and lesser numbers of CD3+ T cells and MHC II+ antigen‐presenting cells, that are reasonably regulated by IL‐10 alterations.^20^

## CONFLICT OF INTEREST DECLARATION

The authors have no conflict of interest to report, beyond disclosures of employment.

## OFF‐LABEL ANTIMICROBIAL DECLARATION

Authors declare no off‐label use of antimicrobials.

## INSTITUTIONAL ANIMAL CARE AND USE COMMITTEE

Approved by Animal Clinical Investigation Animal Care and Use Committee (Chevy Chase, MD).

## HUMAN ETHICS APPROVAL DECLARATION

Authors declare human ethics approval was not needed for this study.

## Supporting information


**Appendix** **S1**: Supporting information
