# Supplementary material for: Leukocyte and cytokine variables in asymptomatic Pugs at genetic risk of necrotizing meningoencephalitis
Source: J Vet Intern Med. 2021 Oct 23;35(6):2846–52. doi: 10.1111/jvim.16293 (PMC8692191; doi:10.1111/jvim.16293)
Supplement: Supplementary file 1 — Appendix S1: Supporting information [file JVIM-35-2846-s001.pdf]

Altered systemic immune parameters in asymptomatic animals at genetic risk for Pug Dog Encephalitis
Supplemental Materials

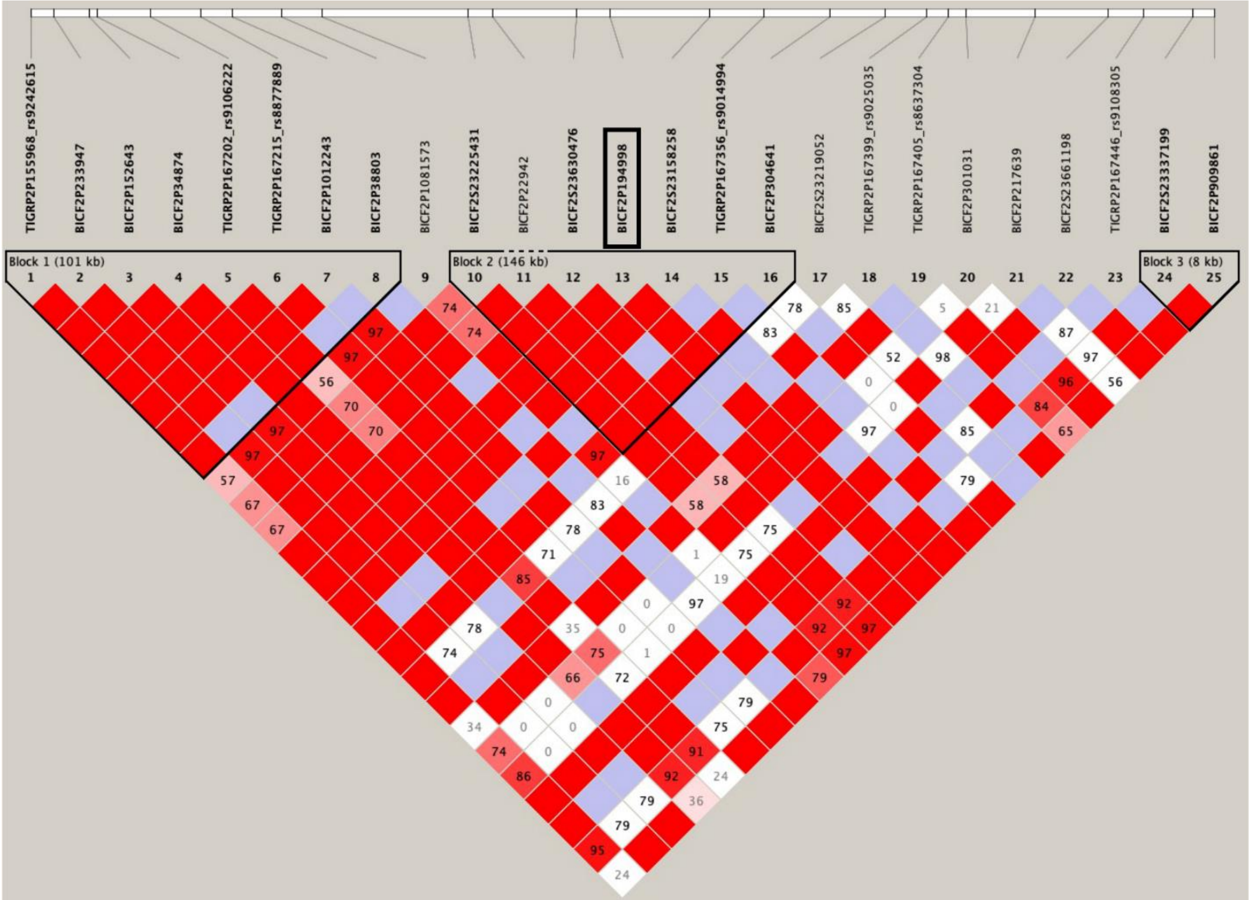

Figure A. Identification of an NME risk-associated haplotype. Shown is the Haploview illustration of the linkage disequilibrium (LD) structure across a 500 kb region centered on the top associated SNP (BICF2P194998, black box) from the Barber et al. pug NME GWAS. The three black outlined triangles indicate high-confidence haplotype blocks (labeled Block 1-3) based on the D' and LOD (logarithm of the odds) determinations for each SNP pair. Blue and white colored diamonds indicate LOD < 2.0 and D' = 1 or < 1 respectively. Shades of pink/red diamonds indicate LOD ≥ 2.0 and D' < 1 while bright red diamonds indicate LOD ≥ 2.0 and D' = 1 (the strongest statistical evidence for linked SNPs). Block 2 contained the top associated SNP and was explored further to identify the haplotypes and their associated frequencies.

Association	BICF2S23225431	BICF2P22942	BICF2S23630476	BICF2P194998	BICF2S23158258	TIGRP2P167356_rs9014994	BICF2P304641	Case Freq. Barber et al.	Control Freq. Barber et al.	P-value Barber et al.	Chi Square Barber et al.	Freq. Current Study
RISK	G	G	A	A	G	A	G	0.886	0.271	1.3E-13	54.862	0.300
PROTECTIVE	A	A	A	T	G	A	A	0.045	0.307	4.0E-04	12.632	0.400
PROTECTIVE	A	A	C	T	A	A	G	0.045	0.211	0.011	6.555	0.150
PROTECTIVE	A	A	A	T	G	G	G	0.000	0.127	0.013	6.185	0.113
PROTECTIVE	G	G	A	T	G	A	G	0.023	0.084	0.158	1.990	0.038

Table A. Haplotype Block 2 SNP components, frequencies, and association statistics. This table indicates the SNPs and their respective genotypes for each of the different haplotypes identified in Block 2. Shown are the frequencies and association statistics for each haplotype in the Barber et al. cohort as well as the frequencies in the cohort under study in this manuscript. Note the single pug NME risk haplotype identified at this locus (top row). The dosing of this haplotype was used to infer NME risk in the current study wherein high-risk animals were determined to be those that carried two risk haplotypes, medium-risk animals carried one risk haplotype, and low-risk animals carried no risk haplotypes.
